# Nanomedicine-based technologies and novel biomarkers for the diagnosis and treatment of Alzheimer’s disease: from current to future challenges

**DOI:** 10.1186/s12951-021-00864-x

**Published:** 2021-04-29

**Authors:** Amanda Cano, Patric Turowski, Miren Ettcheto, Jason Thomas Duskey, Giovanni Tosi, Elena Sánchez-López, Maria Luisa García, Antonio Camins, Eliana B. Souto, Agustín Ruiz, Marta Marquié, Mercè Boada

**Affiliations:** 1 Research Center and Memory Clinic, Fundació ACE. Institut Català de Neurociències Aplicades, International University of Catalunya (UIC), C/Marquès de Sentmenat, 57, 08029 Barcelona, Spain; 2 Biomedical Research Networking Centre in Neurodegenerative Diseases (CIBERNED), Madrid, Spain; 3 Institute of Nanoscience and Nanotechnology (IN2UB), Barcelona, Spain; 4 Department of Pharmacy, Pharmaceutical Technology and Physical Chemistry, Faculty of Pharmacy and Food Sciences, University of Barcelona, Barcelona, Spain; 5 UCL Institute of Ophthalmology, University College of London, London, UK; 6 Department of Pharmacology, Toxicology and Therapeutic Chemistry, Faculty of Pharmacy and Food Sciences, University of Barcelona, Barcelona, Spain; 7 Nanotech Lab, Te.Far.T.I, Department of Life Sciences, University of Modena and Reggio Emilia, Modena, Italy; 8 Umberto Veronesi Foundation, 20121 Milano, Italy; 9 Department of Pharmaceutical Technology, Faculty of Pharmacy, University of Coimbra, Coimbra, Portugal; 10 CEB - Centre of Biological Engineering, University of Minho, Campus de Gualtar, 4710-057 Braga, Portugal

**Keywords:** Alzheimer’s disease, Biomarkers, Polymeric nanoparticles, Lipid nanoparticles, Metal nanoparticles, Nanotechnology

## Abstract

Increasing life expectancy has led to an aging population, which has consequently increased the prevalence of dementia. Alzheimer's disease (AD), the most common form of dementia worldwide, is estimated to make up 50–80% of all cases. AD cases are expected to reach 131 million by 2050, and this increasing prevalence will critically burden economies and health systems in the next decades. There is currently no treatment that can stop or reverse disease progression. In addition, the late diagnosis of AD constitutes a major obstacle to effective disease management. Therefore, improved diagnostic tools and new treatments for AD are urgently needed. In this review, we investigate and describe both well-established and recently discovered AD biomarkers that could potentially be used to detect AD at early stages and allow the monitoring of disease progression. Proteins such as NfL, MMPs, p-tau217, YKL-40, SNAP-25, VCAM-1, and Ng / BACE are some of the most promising biomarkers because of their successful use as diagnostic tools. In addition, we explore the most recent molecular strategies for an AD therapeutic approach and nanomedicine-based technologies, used to both target drugs to the brain and serve as devices for tracking disease progression diagnostic biomarkers. State-of-the-art nanoparticles, such as polymeric, lipid, and metal-based, are being widely investigated for their potential to improve the effectiveness of both conventional drugs and novel compounds for treating AD. The most recent studies on these nanodevices are deeply explained and discussed in this review.

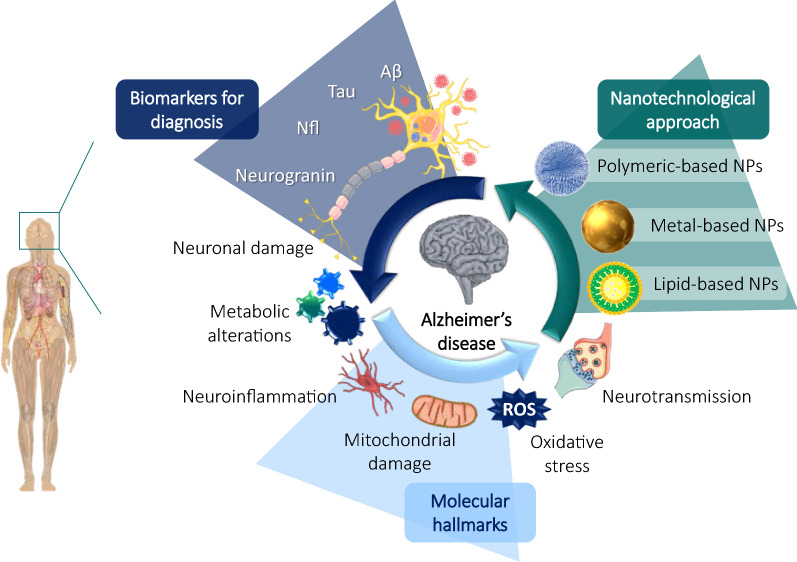

## Highlights

- Alzheimer’s disease is the most common form of dementia worldwide, estimated to be responsible for 50–80% of all cases.
- No currently available treatment can stop the disease progression, and it is expected that AD cases will reach 131 million by 2050.
- The late diagnosis of AD will have a serious impact on health and socio-economic systems worldwide.
- Novel biomarkers, such as NfL, SNAP-25, Clusterin, MMPs, and YKL-40, among others, are being studied alongside commonly used biomarkers as potential new diagnostic tools.
- Nanomedicine has aroused much interest in the last decade and has shown promising results for improving drug targeting and delivery to the brain.
- Polymeric-, lipid-, and metal-based nanoparticles are the most commonly investigated nanodevices in the field of dementia.
- State-of-the-art nanoparticles are emerging as promising tools to improve the diagnosis and treatment of AD.

## Introduction

**Fig. 1:**
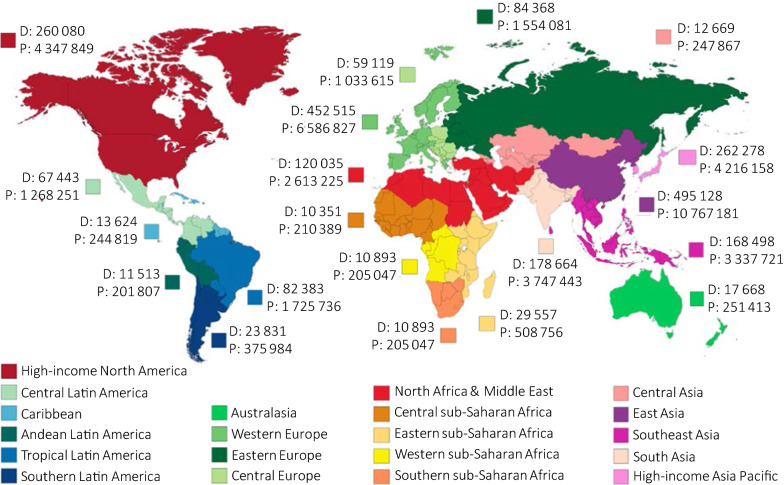
Global deaths (D) and prevalence (P) for Alzheimer’s disease and other dementias in 2016. Data are n (95% UI). UI = uncertainty interval. Data extracted from the Global Burden of Disease Study of Alzheimer’s disease and other dementias 2016 [1]

In the past few decades, the number of people living with dementia has increased exponentially all around the world, mainly due to aging populations and improvements in quality of life. The latest *Global Burden Disease Study* estimated that the number of cases of dementia more than doubled from 1990 to 2016 and indicated population growth and aging as the main drivers of this increase (Fig. 1) [1].

Alzheimer’s disease (AD) is the most common form of dementia worldwide, estimated to constitute up to 50–80% of cases [1]. Undoubtedly, AD will critically burden economies and health systems since cases are expected to reach 131 million by 2050 [2]. AD is commonly diagnosed symptomatically through the occurrence of significant memory loss, global cognitive decline, and the impairment of daily life activities. Later in the course of the disease, the breakdown of physical functions, such as walking, swallowing, and general movement, ultimately leads to death [3]. Dementia was the fifth-leading cause of death in 2016 [1].

AD can be classified depending on the onset of the first symptoms. Approximately 1–6% of all cases are categorized as early-onset AD (EOAD), which manifests before the age of 65. By contrast, late-onset AD (LOAD) is characterized by the occurrence of symptoms at an age greater than 65 years and accounts for around 90% of cases [4]. EOAD differs from LOAD in many aspects. Prominently, EOAD has a more aggressive course of disease progression, greater delay to diagnosis, lower cognitive reserves, lower incidence of diabetes, obesity, and circulatory disorders, relatively greater deficits in attention, executive functions, praxis, and visuospatial functions, lower frequency of the APOE ε4 allele, greater white matter changes, and a higher burden of neuritic plaques and neurofibrillary tangles [5].

In general, AD is defined as a multifactorial disease in which genetic, environmental, behavioral, and developmental components critically influence its pathogenesis, with age being the most important risk factor. Although most cases of AD are sporadic, rare cases (< 1%) have a genetic component (“Familial AD”, FAD) with a few hundred families identified worldwide. These cases usually have an early onset at young ages (40–50). The three known genes that cause FAD, with an autosomal dominant inheritance, are the amyloid precursor protein (APP), presenilin-1 (PS1), and presenilin-2 (PS2) genes, all of which are involved in the processing or production of Aβ [6]. Our understanding of the pathophysiology of AD is constantly changing [4]. The overproduction and accumulation of amyloid-β (Aβ) peptide and hyperphosphorylated tau (p-tau) protein are the two most common hypotheses for AD pathogenesis, but recent findings have demonstrated that chronic oxidative stress, hormone imbalance, mitochondrial dysfunction, inflammation, calcium mishandling, mitotic dysfunction, genetic components or blood–brain barrier (BBB) dysfunction also likely play key roles in the disease process [7, 8]. Similarly, emerging studies have also reported that oligodendrocytes act as antigen-presenting cells and produce immune molecules. Likewise, the activation of both astroglia and oligodendrocytes, mainly due to BBB dysfunction and general toxic species produced in AD, occurs widely in AD [9]. In neuroinflammation processes, oligodendrocytes express many factors known to activate astrocytes. In response to these factors, astrocytes and oligodendrocytes secrete immune factors, underscoring the possible immune function of these cells. IL-1β, IL17 tumor necrosis factor-α (TNF-α), interferon-γ (IFN-γ), and fibroblast growth factor-2 (FGF-2) are some of these secreted cytokines that induce pro-inflammatory effects [9]. Regardless of all these changes, neuronal death is the ultimate invariable outcome, which then drives the typical neurodegeneration of AD.

Currently there are only four FDA approved treatments for AD, and these are linked mainly to the two molecular pathways involving the accumulation of Aβ peptide and neurofibrillary tangles (NFT) of p-tau protein [10]. However, none of these drugs stops disease progression or cures AD, highlighting the need for additional treatment approaches. The discovery of novel biomarkers is hoped to deliver earlier AD diagnosis and could also support the identification of additional molecular targets, potentially leading to new treatments.

Identifying the pathophysiological processes involved in AD and the best biomarkers to detect them is critical for the development of novel cures. In addition, efficiently and specifically delivering the diagnostic and therapeutic molecules to the sites of interest in these processes is critical. Nanoparticles (NPs) have enabled great strides towards the delivery, treatment and diagnostics of diseases, mainly due to their various chemical characteristics and their propensity for chemical modification to modulate and refine required properties [11, 12]. NPs’ core constituents comprise a wide variety of materials, such as lipids, polymers, and metals, that can encapsulate molecules with different chemical natures. In addition, these carriers promote the protection and delivery of bioactive molecules, which can reduce their potential toxicity and, in turn, enhance their solubility, stability, biodistribution, and pharmacokinetics. Molecules encapsulated in NPs range from small molecules, peptides, and proteins to genetic material [13]. Crucially for chronic CNS diseases, NPs have the demonstrated capacity to deliver molecules to hard-to-reach tissues, such as the CNS where the crossing of the BBB and the release of drugs with controlled kinetics for long-term treatments are required [11, 14].

In this review, we investigate and describe well-established AD biomarkers and highlight recently discovered ones that could be potential diagnosis tools. In addition, we explore the most recent nanomedicine-based technologies used to target drugs across the BBB and increase CNS delivery of these active molecules.

## Novel biomarkers in the diagnosis of Alzheimer’s Disease

AD was first described in 1906 [15]. For a long time after that, firm diagnosis was made when signs of memory loss and cognitive decline were already significantly advanced. Nowadays, many observations indicate that the pathophysiological alterations of AD in the brain begin decades before the onset of clinical symptoms of dementia. Historically, AD patients were classified into three clinical categories: cognitively unimpaired (CU), mild cognitive impairment (MCI), and dementia patients [16]. AD is now recognized as a continuum of neurological decline that can be identified and staged through a combination of neuropathological findings and in vivo biomarkers [17, 18]. This has led to a paradigm shift in the diagnosis of AD, opening new windows of opportunities for early treatment in the preclinical stages. In that context, the *National Institute on Aging* and the *Alzheimer’s Association* have recently updated diagnostic criteria, with a clear shift from a clinical to a biological definition of AD [19].

Biomarkers now have key importance in the robust diagnosis of AD. Biomarkers are quantifiable molecules or processes that can be related to the biological alterations and/or pharmacologic responses to a therapeutic intervention for a specific disease [18, 20]. An ideal biomarker should be specific, sensitive, predictive, accurate, robust, inexpensive, and ideally non-invasive and measurable in common biological fluids such as serum, saliva and/or urine [21, 22]. For AD, cerebrospinal fluid (CSF) is considered the optimal biological source for biomarker assessment, since its direct contact with the interstitial fluid where the brain is immersed reflects the pathophysiological changes of AD progression in real time [18, 23]. For AD biomarkers, specificity and sensitivity of more than 80% are needed to be considered a reliable biomarker [18]. Notably, not all patients with cognitive impairment develop Aβ plaques or tau neurofibrillary tangles (NFTs), and vice versa. Furthermore, biomarkers have different uses and interpretations depending on the degree of the cognitive deterioration observed. Thus, the use of biomarkers has allowed the identification of prodromal AD in MCI subjects and the confirmation of AD in individuals with dementia [18].

**Fig. 2:**
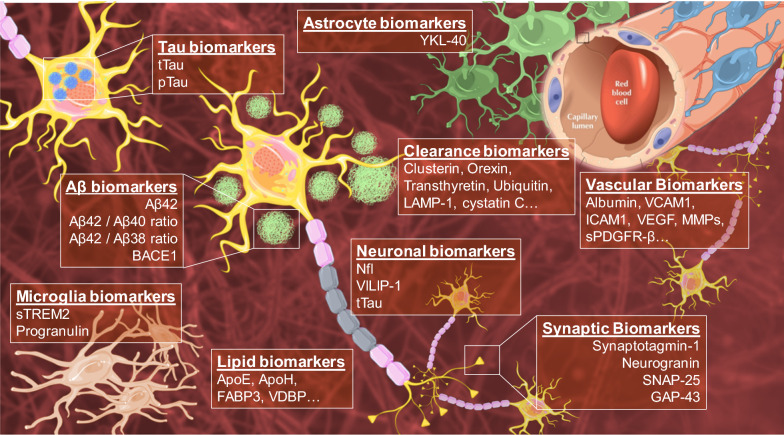
Conventional and novel biomarkers related to molecular alterations and physiopathological changes of AD

Several biomarkers are currently being used in a complementary way with conventional neuropsychological tests and routine neurological exams in the diagnosis of AD. The parameters and diagnostic implications of these conventional biomarkers have recently been reviewed and updated. Furthermore, new molecules, related to different pathophysiological pathways involved in the development of AD, are being identified as potential new biomarkers for the early diagnosis of this disorder (Fig. 2) [24–27].

### Conventional AD biomarkers in CSF/plasma

#### Aβ-related biomarkers

In AD pathogenesis, the production of Aβ peptides is increased, while their clearance is reduced. Aβ1-40 and Aβ1-42 fragments predominantly form, promoting the aggregation of these peptides and thus leading to the formation of senile plaques [28]. Aβ42 is one of the most toxic isoforms of the Aβ peptide and is also one of the most widely accepted biomarkers of AD diagnosis [29, 30]. Aβ42 quantification in CSF allows the identification of AD in its preclinical stage, and possesses high diagnostic value with clear specificity for AD over other neurodegenerative diseases. However, absolute Aβ42 levels vary due to a variety of factors, particularly interindividual differences [29]. To account for these, the ratio between Aβ42/Aβ40 is also commonly used. Aβ40 concentration is 10-times higher than Aβ42 in CSF and its level does not usually vary in AD [31]. Similarly, the Aβ42/Aβ38 ratio is emerging as a potential predictive tool with comparable power to the Aβ42/Aβ40 ratio [32–34].

Likewise, as with other disease biomarkers, current efforts are also focused on improving Aβ42 detection in blood samples [28, 29]. CSF extraction through lumbar function is an invasive procedure that is only permitted for certain patients. It is often not feasible, e.g., for patients with low back injuries, increased intracranial pressure due to a space-occupying lesion, coagulopathy, or infected skin over the puncture site [35, 36]. Immunoprecipitation coupled with mass spectrometry, ELISA, and protein misfolding cyclic amplification assays are possible techniques for detecting blood Aβ42 [29, 37]. Recently, a blood analytical test for the detection of plasma Aβ by mass-spectrometry gained approval by regulators at the Centers for Medicare & Medicaid Services under the Clinical Laboratory Improvement Amendments protocol [38]. Researchers predict that the next tests to be approved are plasma p-tau181 and p-tau217, as these tests will be more robust since they possess a larger dynamic range. Indeed, phosphorylated fragments of tau increase by as much as tenfold in the blood as AD progresses, whereas the Aβ42/40 ratio falls by about 15 percent at most [38].

BACE1, the key enzyme that initiates the formation of Aβ peptide [39], has increasingly been studied as a potential AD biomarker. BACE1 can be measured in CSF, but there is controversy regarding its predictive value. Some studies have shown increased levels and protein activity of BACE1 in AD patients and that this is a good predictor of the progression of MCI patients [40, 41]. However, more recent studies have reported no changes in CSF BACE1 levels among controls, MCI and AD patients [42–44].

#### Tau-related biomarkers

It is widely accepted that hyperphosphorylated tau (p-tau), accumulating as intracellular NFTs, is the other main pathological hallmark of AD, together with Aβ plaques [29, 45]. However, in many neurodegenerative diseases, collectively known as "tauopathies", p-tau NFTs are found that differ in their phosphorylated residues [29, 46]. The diagnostic value of total tau (t-tau) levels for differentiating AD from normal aging is well described. T-tau has been considered a relevant biomarker of neuronal injury and thus is not a specific marker of AD; it is also found to be elevated in other neurological conditions such as Creutzfeldt-Jakob’s disease, frontotemporal dementia with parkinsonism, and Pick’s disease, among others [47]. P-tau (specifically tau phosphorylated on threonine 181, p-tau181) has been described as a more specific biomarker for AD since p-tau is present at normal levels in most other neurodegenerative disorders, but not in AD [29]. In clinical practice, the correlation between both t-tau and p-tau in CSF is important for differentiating among several dementia types [48].

Levels of tau deposition display a stronger correlation to cognitive decline than does Aβ in AD patients [49]. However, longitudinal studies have revealed that CSF tau levels vary depending on the different stages of the disease [49, 50]. Several recent studies have tried to elucidate the prognostic value of tau in early-onset AD and as a biomarker to monitor drug responses, but further investigation is required [29].

As with Aβ, progress is also being made regarding the predictive value of tau in blood samples thanks to ultrasensitive immunoassays techniques. Some studies have already identified higher plasma and/or serum p-tau181 levels in AD patients versus controls using innovative techniques such as Simoa®, immunomagnetic reduction, and label-free real-time surface plasmon resonance [51–53]. Interestingly, recent findings have shown the predictive value of plasma p-tau217 for discriminating AD from other neurodegenerative diseases, specifically as an accurate predictor of β-amyloidosis at asymptomatic and symptomatic stages [54–57]. In these studies, p-tau-217 and p-tau-181 were highly specific for amyloid plaque pathology, as p-tau-217 measurement was still more specific to amyloid status than p-tau-181 [54–57]. Collectively, these findings raise the possibility for a novel, highly sensitive, and specific biomarker based on circulating tau, with improved diagnostic accuracy. Further studies are needed to validate these findings in unselected and diverse populations and determine its potential role in clinical practice [54–57].

### Novel AD biomarkers in CSF/plasma

#### Neuronal damage-related biomarkers

Other molecules and cellular structures have been suggested as potential biomarkers of neuronal damage. Among these, neurofilaments, specifically neurofilament light chain (NfL), have emerged as promising biomarkers. After axonal and/or neuronal damage, NfL leaks into CSF and increases to detectable levels [58].

Although NfL is not specific to AD, it has been shown to be intimately linked to neurodegenerative diseases, and its predictive value increases in combination with other biomarkers [59]. However, one of the benefits of NfL compared to other biomarkers is the high correlation of its levels in CSF and blood [60]. Moreover, similarly to Aβ, increased levels of NfL can be detected in the early stages of autosomal dominant AD, even before the onset of first symptoms [61]. In addition, a recent 18-month trial with mild AD patients showed that NfL, as a Tau-independent marker of axonal degeneration, had a stronger association with clinical scales than did t-tau [62]. Thus, the ability of NfL to detect changes before clinical manifestations occur reveals its promise as a diagnostic biomarker.

Visinin-like protein 1 (VILIP-1), a calcium sensor protein highly expressed in neurons, is another protein that could act as a biomarker of neuronal-injury in AD [18, 29]. This is supported by the strong correlation between increased levels of both VILIP-1 and tau in the CSF of AD patients [63, 64]. Importantly, some studies have reported the predictive value of VILIP-1 for atrophy rates and cognitive decline, indicating that this protein could help identify MCI patients that will progress to AD [64]. In blood samples however, the predictive value of VILIP-1 remains uncertain [27, 29].

#### Neuroinflammation-related biomarkers

Inflammatory processes play important roles in the pathogenesis of AD. The activation of glial cells, the resident immune cells of the CNS, is well described for different neurodegenerative diseases, especially AD [65, 66]. Historically, it was accepted that neuroinflammation, initiated primarily as a reaction to Aβ and p-tau neurotoxicity, triggered the production of neurotoxic molecules (e.g. ROS, glutamate or inflammatory cytokines). This pattern is reproduced in a continuous molecular feedback loop, which has been described to be more pronounced in late stages of AD [67]. However, recent evidence also reveals the neuroprotective role of microglia and astroglia in earlier stages of AD development [67, 68]. When the deposition of Aβ plaques appears, glial cells create an immune barrier that surrounds and isolates these senile plaques. This process protects the axons adjacent to Aβ plaques from Aβ neurotoxicity [50, 60]. Recent genetic studies have found correlations between the deficiency of microglia encapsulation toward Aβ deposits and an increased risk of late-onset AD [53, 58]. In addition, an inverse correlation between neuroinflammation in the brain cortex and plasmatic NfL levels has been reported [69, 70]. In normal aging, serum levels of NfL show a nonlinear increase from 60 years old in both males and females [71]. However, low plasma NfL levels are associated with raised cortical microglial activation, suggesting that inflammation acts to protect prodromal AD [70].

Consequently, neuroinflammation biomarkers may be of great interest for the early diagnosis of preclinical AD. YKL-40, also known as human cartilage glycoprotein 39 and predominantly related to astroglial proteins, is one of the most studied neuroinflammation-related biomarkers [72]. YKL-40 is not only upregulated in AD, but in other diseases in which inflammation plays an important role [72]. Longitudinal studies and recent meta-analyses have reported that CSF YKL-40 levels are higher in AD patients compared with controls, increase throughout the disease progression and are positively correlated with neuronal-injury biomarkers, mainly in the preclinical stages of AD [30, 64]. However, YKL-40 has not yet shown consistent results in blood samples.

Progranulin, a glycoprotein mainly secreted by activated microglia, is involved in the modulation of neuroinflammation [73]. This protein has aroused much interest since its loss-of-function has been closely correlated with some types of frontotemporal lobar degeneration [29, 74]. However, although a cross-sectional study reported increased CSF progranulin levels in autosomal-dominant AD and late-onset AD, progranulin from CSF or blood is currently not validated as diagnostic markers [75, 76].

Another neuroinflammation-related biomarker that has attracted attention is the triggering receptor expressed on myeloid cells 2 (TREM2), which has an arguably controversial role in AD development. This transmembrane protein belongs to the immunoglobulin family and is expressed in microglial cells [77]. TREM2 is involved in many biological processes, such as migration, proliferation, cytokine release, APOE binding, or sealing of Aβ plaques [29, 68]. Moreover, a rare missense mutation, predicted to lead to an R47H substitution in TREM2, has been linked to a significant risk of AD in Iceland [78]. It was also reported that the soluble ectodomain of TREM2 (sTREM2) is released into the extracellular space and may serve as a CSF biomarker [29]. However, to the best of our knowledge, the biological role of sTREM2 is unclear, particularly in neuroinflammation. Some studies have shown that hyperstimulation of the TREM2 pathway correlates to the attenuation of microglial activation [79–81]. However, further studies are needed to provide firm mechanistic evidence.

Taken together, most of these inflammation-related biomarkers are not specific to AD and have thus been proposed as “neuroinflammation-tracking assistants” that can provide a more solid diagnosis together with specific AD biomarkers. Additionally, they could be used to identify AD patients who would benefit from novel microglia-targeted treatments.

#### Synaptic dysfunction-related biomarkers

Synaptic degeneration is another hallmark of early AD pathology and appears to closely correlate with cognitive decline [82]. This event is associated, in turn, with the neurotoxic effects of Aβ and tau species and glutamatergic excitotoxicity, which lead to alterations in axonal transport that later promote dendritic alterations and eventually neuronal loss [83]. Many molecules involved in synaptic degeneration have been identified as potential biomarkers and have been divided into pre- and post-synaptic biomarkers.

Growth-associated protein 43 (GAP-43) belongs to the group of potential presynaptic biomarkers [27, 29]. This protein is involved in synaptogenesis and neuronal development and is mainly expressed in the cortex, hippocampus, olfactory bulb, and cerebellum. In fact, three decades ago, the correlation between an increased density of neurons containing NFTs and decreased levels of GAP-43 in the brain and cerebellar cortex of AD patients had already been reported [84]. More recently, the significant elevation of CSF GAP-43 in AD patients has been reported [85]. Interestingly, this increase also correlates with the amount of Aβ and p-tau found in the cortex, amygdala and hippocampal structures [85]. These findings indicate that GAP-43 is a promising biomarker for AD in CSF. However, its usefulness in blood samples has not yet been well described.

Synaptosomal-associated-protein-25 (SNAP-25) is a newly discovered potential biomarker related to pre-synaptic damage. Recent studies reported that CSF SNAP-25 increases in MCI and AD, which is associated with the rate of hippocampal atrophy and cognitive decline [86]. Moreover, CSF SNAP-25 levels are also substantially higher in MCI patients who are APOE ε4 carriers, compared to non-carriers. These findings suggest the potential relevance of SNAP-25 to predict the conversion from MCI to AD [87]. SNAP-25 may even have relevance as a blood biomarker. Agliardi et al*.* showed that SNAP-25 can be detected in serum, where it appears in neuron-derived plasma exosomes, which are extracellular vesicles involved in intercellular communication. Serum levels of SNAP-25 are lower in AD patients compared to healthy controls [88]. Although further studies are needed, these findings indicate the potential use of SNAP-25 as a pre-synaptic injury-related biomarker.

Synaptotagmin-1 was one of the first proteins detected in the CSF of early-onset AD patients [89]. Synaptotagmin-1 is a pre-synaptic vesicle protein involved in the maintenance of correct synaptic transmission and cognitive function. Recent studies reported increased CSF Synaptotagmin-1 levels in both MCI and AD patients compared to healthy controls [90], indicating that Synaptotagmin-1 is a potential CSF biomarker of both AD and conversion from MCI to AD.

Neurogranin (Ng) is a post-synaptic protein that regulates calcium signaling and synaptic plasticity mainly found in the dendritic spines of the amygdala, hippocampus, and caudoputamen [29, 82]. High CSF Ng levels have been reported to predict future cognitive decline specific to AD pathogenesis, with better specificity than p-tau [91]. Furthermore, several studies have shown elevated CSF Ng levels in MCI and prodromal AD patients [91, 92]. Interestingly, a recent study carried out by Kirsebom et al. identified an elevated CSF Ng/BACE1 ratio as a predictor of very early cognitive decline in AD. Specifically, elevated Ng/BACE1 is associated with lower hippocampal and amygdala volumes of MCI patients compared to healthy controls [93]. All these findings highlight Ng as one of the most relevant biomarkers related for synaptic dysfunction in AD. The scientific evidence to date has not clarified the predictive value of blood Ng as an AD synaptic-damage biomarker. In addition, further studies are needed to define the specific range of Ng values for diagnoses of different stages of AD.

#### BBB dysfunction-related biomarkers

Cerebrovascular disease and AD share multiple risk factors. Substantial evidence suggests that vascular dysfunction is the earliest event in the pathogenic development of late-onset AD [94]. Vascular dysfunction, associated with aging, causes reduced oxygen, glucose, and nutrient supply to the brain, which directly damages not only the parenchymal cells, but also the BBB structure. This in turn promotes the overproduction of ROS, nitric oxide, and inflammatory cytokines in response to the neurotoxic effects of vascular dysfunction, which contributes to a vicious circle of global neurotoxicity affecting both BBB dysfunction and AD pathogenesis [94]. The standard biomarker of BBB dysfunction measured in clinical practice is the CSF/serum ratio of albumin [95]. Although the role of albumin and its predictive value are still controversial, many efforts have been made to exploit the usefulness of this biomarker. Other molecules have also emerged as potential biomarkers of BBB-dysfunction, such as VCAM-1, ICAM-1, MMPs, VEGF, PIGF, sPDGFR-β, and tight junction proteins (such as claudins and occludin) [25, 29]. However, further studies are needed to determine their predictive value in CSF, as well as their correlation in peripheral blood.

#### Lipid metabolism-related biomarkers

Brain tissue is highly enriched in lipids. Lipid metabolism is decisive for the synaptic activity, neuronal survival, and immune responses of glial cells, and AD pathogenesis is accompanied by continuous changes in brain lipid patterns [96]. Thus, proteins related to lipid transport and metabolism in the CNS have been proposed as potential biomarkers of AD pathogenesis. Undoubtedly, ApoE is the prototypical protein involved in lipid homeostasis and AD development. The expression of the ApoE ɛ4 allele is well known as the strongest genetic risk factor for AD [27, 97]. Therefore, CSF ApoE levels have been investigated as AD progression biomarkers and for the differential diagnosis of AD and other neurodegenerative disorders [27]. However, further studies are needed to validate its potential predictive value in blood samples. Similarly, heart fatty acid-binding protein (FABP3), ApoH, and vitamin D-binding protein are related to lipid metabolism and increased levels of them have been found in the CSF of AD patients. Moreover, these have been proposed for the differential diagnosis of AD from Lewy body dementia, Parkinson’s disease, and other dementias [27].

#### Neurotoxins clearance-related biomarkers

The clearance of toxic metabolites from the brain is essential for healthy function. Since the dysregulation of clearance mechanisms has been identified as a direct cause of AD development, proteins related to the removal of cerebral metabolic waste, Aβ and p-tau peptides, and reactive oxidative species have been suggested as candidate biomarkers [27, 98]. Clusterin, Orexin and Transthyretin, which are involved in the clearance of partially unfolded proteins and Aβ peptides, have thus been described as potential biomarkers [27]. Interestingly, clusterin, also called APOJ, is also related to lipid transport, inflammation, and chaperone activities. Similarly, LAMP-1, carboxypeptidase E, cystatin C, and ubiquitin CSF levels are increased in patients with AD and are emerging as potential neurotoxin clearance-related biomarkers [27, 99].

#### Metal ion homeostasis-related biomarkers

Metal ions have been widely described as potential targets for the diagnosis and treatment of AD. The abnormal accumulation of metal ions, like zinc, copper, and iron, in the brain has been closely related to the overproduction of Aβ peptide and p-tau, and the accumulation of senile plaques and NFTs [100]. Likewise, abnormalities in metal-binding proteins are a key factor in promoting the erroneous distribution and deposition of metal ions in the brain [100]. Some of the mechanisms by which metal ions promote these abnormalities are the induction of oxidative stress, autophagy dysfunctions, the disruption of endoplasmic reticulum and mitochondria structures, activation of β- or γ-secretases, inhibition of α-secretase, and activation of protein kinases such as cyclin-dependent protein kinase-5 (CDK5), glycogen synthase kinase-3β (GSK-3β), mitogen-activated protein kinases (MAPKs), etc. [100]. Moreover, all these alterations enhance the abnormal brain deposition of metal ions and further dysregulate their homeostasis. Therefore, these metal ions have been proposed as potential biomarkers for tracking AD progression. Likewise, adjusting metal balance may be a potential treatment for AD pathologies and is a promising path for future research.

## Molecular strategies for the therapeutic approach to Alzheimer’s disease

### Amyloid-β strategies

As explained above, the overproduction and accumulation of Aβ lead to several neuropathological processes that trigger neuronal death, which translates into the memory loss and cognitive disorders typical of this disease [28, 101]. α-secretase is the main enzyme in amyloid-precursor protein (APP) metabolism and its action is followed by that of γ-secretase in physiological conditions. Amyloidogenic processing occurs when an alternate enzyme, β-secretase, acts instead of α-secretase [102]. Generally, Aβ-based therapies target several aspects of APP metabolism [101]. Likewise, Aβ-binding to several receptors has been related with some of its neurotoxic mechanisms (Table 1) [103]. Thus, therapeutic targeting to these proteins has been proposed to decrease amyloidogenic APP processing and Aβ-related toxicity.

**Table 1:** Main receptors and their implications in Aβ binding-mediated neurotoxic effects [103]

Receptor	Localization	Proposed mechanisms
NMDAR	Postsynaptically located on dendrites and dendritic spines	Impairment of NMDAR activity: removal from the cell surface and triggering of synaptic depression signalling pathways Increase of NMDAR function: AβOs induce neuronal oxidative stress through an NMDAR-dependent mechanism AβOs bind to NMDAR →excessive activation of NMDAR →inflow of Ca^2+^ to neurons →excitotoxicity
AMPAR	Hippocampal pyramidal neurons and dendritic spines	AβOs → synaptic dysfunction by inducing calcineurin-dependent internalization of AMPAR
PrPC	Brain neurons and spinal cord	Initial interaction of AβOs with PrPC on the neuronal surface which leads to: Disturbed regulation of BACE1 activity Inhibition of elongation of Aβ fibrils Intracellular Ca^2+^ increase in neurons via the complex PrPC-mGluR5 →impairment of synaptic plasticity
mGluR5	Hypothalamus and cortex	Complexes of AβOs with PrPC generate mGluR5-mediated influx of Ca^2+^ in neurons → excitotoxicity AβO-PrPC-mGluR5 complexes signalling pathway involved in dendritic spine loss
β2ARs	Locus coeruleus, hippocampus and cortex	AβOs induce the degradation of β2ARs, which leads to: Enhanced γ-secretase activity → Aβ plaque formation Reduction of neurogenesis Reduction of the levels of synapse-associated proteins such as synaptophysin, synapsin 1, and PSD-95
α7nAChR	Septo-hippocampal region and cortical neurons	Aβ42 binds to α7nAChR → loss of cholinergic neurons in the brain → receptor internalization and intracellular accumulation of Aβ
IR	Choroid plexus, olfactory bulb and regions of the striatum and cerebral cortex	AβOs bind to neuronal IR → impaired insulin signalling and brain insulin resistance → elevated Aβ production and reduced AβO clearance → Aβ deposits in the brain → neuronal damage
p75NTR	White matter brain *regions and* spinal cord	AβOs bind to membrane p75NTR → formation of annular amyloid pores and ion channels → induction of aberrant cytoskeletal changes in dendritic spines AβOs bind to IGF-1R → phosphorylation of IGF-1R → induced p75NTR expression → cell death by fibrillary form of Aβ
ILR	Hippocampus and surface of B lymphocytes, dendritic cells, natural killer cells, macrophages, granulocytes, mast cells, etc	AβOs bind to PirB → impartment of synaptic plasticity → disruption of hippocampal long-term potentiation → Aβ-induced deficits of memory AβOs bind to FcγRIIb → AβO-induced inhibition of long-term potentiation → Aβ-mediated neuronal dysfunction
TREM2	Surface of immune cells of myeloid origin	AD-associated TREM2 mutations → reduction of AβOs binding and degradation of Aβ → microglial depolarization, induction of K^+^ current into cells as well as increased cytokine expression and secretion, cells migration, proliferation, apoptosis, and morphological changes of microglia
Eph4A EphB2	Hippocampal neurons	AβOs reduce Eph receptor expression, promote its endocytosis and its degradation in the proteasome, which leads to: Loss of dendritic spine Synaptic dysfunction Increase of synaptoneurosomes Impairment of NMDAR functioning and cognitive deficits
RAGE	Blood–brain barrier	Expression of RAGE is increased in the AD brain → RAGE is responsible of Aβ influx from plasma to BBB → increase of free Aβ fraction in plasma
LPR2	Choroid plexus epithelium and ependymal cells covering the brain ventricles	Aβ alone did not bind directly to LRP-2, whereas complexes of Aβ-40 with ApoJ are able to react with LRP-2 → clearance of Aβ
VDR	Broadly expressed in all brain regions	1,25-(OH)2D3 binds to VDR → increase the expression of amyloid transporters (i.e. LRP-1) → increase of transport of Aβ across the BBB → Aβ clearance
SIRT1	Predominantly located in the nucleus, but also in the cytosol of neurons of the hippocampus and hypothalamus	SIRT1 deficiency has been described to be responsible for the increased risk of insulin resistance, obesity and diabetes, which in turn are risk factors of AD SIRT1 deficiency has been described to be involve in the reduction of normal cognitive function and synaptic plasticity Reduction of SIRT1 → reduction of α-secretase activity → enhancement of amyloidogenic processing of APP

AβOs, amyloid-β oligopeptides; α7nAChR, Acetylcholine Receptor; AMPAR, α-amino-3-hydroxy-5-methyl-4-isoxazolepropionic acid receptor; β2ARs, β2-Adrenergic Receptors; LPR2, lipoprotein-related protein 2; mGluR5, Metabotropic Glutamate Receptor 5; EphA4, EphB2, Tyrosine Kinase Ephrin Receptors; FcγRIIb, Fragment crystallizable gamma receptor II b; IGF-1R, insulin-like growth factor 1 receptor; ILR, Immunoglobulin-Like Receptors; IR, Insulin Receptor; NMDAR, N-methyl-D-aspartate receptor; p75NTR, p75 Neurotrophin Receptor; PirB, paired immunoglobulin-like receptor B: PrP^C^, Cellular Prion Protein; RAGE, Receptor for Advanced Glycation Endproducts; SIRT 1, Sirtuin 1; TREM2, Triggering Receptor Expressed on Myeloid Cells 2; VDR, vitamin D receptor

In this context, recent pre-clinical studies have suggested that epigallocatechin-gallate (EGCG), the most abundant polyphenol in green tea, induces α-secretase activity and improves non-amyloidogenic APP processing [104]. Likewise, the effect of a one-year treatment with EGCG on cognitive Aβ biomarkers, as well as metabolomics, microbiota, saliva, plasma, and urine, is being evaluated in a randomized, double-blind clinical trial in 200 subjects (NCT03978052) This study aims to demonstrate the potential prevention of cognitive decline in patients with positive ApoEε4 with subjective cognitive decline after a multimodal intervention with EGCG [105]. The estimated study completion date is September 2021, when the first results will reveal the potential of this drug for AD.

Etazolate is another compound that has been shown to possess neuroprotective effects in AD. It is a pyrazolopyridine with anxiolytic-like properties that selectively modulates the GABA_A_ receptor. Marcade et al. demonstrated that Etazolate also promotes neuroprotection via the α-secretase pathway, leading to an induction of sAPPα, which is a neurotrophic and precognitive molecule [106]. Thus, modulators of the GABA_A_ receptors could offer a promising opportunity for the treatment of AD.

The active enantiomer of phenserine, which directly decreases levels of APP by interacting with APP mRNA, has also been found to be effective in enhancing α-secretase and AChE activity [4]. However, other secretase-targeting drugs have not succeeded. For instance, tarenflurbil, the active enantiomer of flurbiprofen, ELND006 and Semagacestat are all γ-secretase inhibitors developed to reduce Aβ levels that failed in clinical trials because of their significant adverse effects, and the absence of results extrapolatable from pre-clinical models to patients [4].

Modulating Aβ transport is another therapeutic approach to AD. Apolipoproteins have an important role in Aβ transport and metabolism since they regulate the movement of Aβ peptides between the periphery and CNS. ApoEε4 increases the passage of Aβ from blood to the brain through the low-density lipoprotein receptor-related protein (LRP) [107, 108]. The peripheral administration of soluble LRP has been proposed as a promising treatment for AD by increasing Aβ efflux from the brain to peripheral blood [109]. Similarly, increasing the Aβ clearance has been studied as a therapeutic option for AD. Some proteases, such as metalloproteinase 9, neprilysin, and insulin-degrading enzyme, can degrade Aβ plaques. The levels of these enzymes decline in AD and this could contribute to Aβ accumulation and senile plaque formation. The activation of these types of enzymes has also been proposed as a therapeutic approach to AD [109].

Therapeutically decreasing Aβ aggregation is one of the most explored routes to interfere clinically with AD progression. Drugs like tramiprosate, EGCG, ELND005, and melatonin have been studied in pre-clinical and clinical trials because of their demonstrated inhibition of the aggregation of Aβ peptides and dissolution of pre-formed fibrils [4]. Likewise, the use of monoclonal antibodies against Aβ aggregation has been explored. For instance, aducanumab has been shown to enter the brain, bind parenchymal Aβ, and reduce soluble and insoluble Aβ in a dose-dependent manner in both transgenic mouse models and patients with prodromal or mild AD [110]. Aducanumab is currently being investigated in a phase III clinical trial (NCT01677572) that is expected to provide compelling support for the amyloid hypothesis. However, the FDA’s advisory committee recently expressed concern about the biostatistical and neurologic results of this trial. Moreover, experts agreed that the aducanumab efficacy data were weak and pointed to inconsistencies in the data that came from two futility-stopped Phase 3 trials and one Phase 1b trial. Thus, the committee deemed the evidence premature for approval and recommended a confirmatory trial. The decision now rests with the FDA, which is predicted to decide by June 2021 [111].

Boada et al. investigated the effects of plasmapheresis with albumin replacement, plus intravenous immunoglobulin and obtained promising results in terms of disease progression in a multicenter, randomized, blinded and placebo-controlled, parallel-group, phase IIb/III clinical trial (AMBAR) of mild-to-moderate AD patients (NCT01561053) [112, 113]. The molecular basis of this innovative therapeutic approach was the uptake and clearance of blood Aβ by replaced albumin, thereby promoting the transport of Aβ peptides from CSF to plasma, which in turn reduces the Aβ burden by restoring the normal balance of Aβ between brain and blood. The trial obtained promising results in terms of disease progression, indicating that this approach is an important development in AD therapeutics [114].

### Tau-based strategies

Targeting AD as a tauopathy is another main therapeutic approach. Soluble tau is found in neuronal cells and plays a dominant role in axonal growth and neuronal development [115]. Its importance lies in its role in regulating and stabilizing microtubules, essential structures of the cell cytoskeleton. While under physiological conditions, the phosphorylation and de-phosphorylation of tau are in equilibrium, thus maintaining its ability to bind to microtubules; in pathological situations, its hyperphosphorylation generates insoluble filaments in the form of tangles, which leads to synaptic dysfunction and neuronal degeneration [115]. Several studies have also shown a relationship between the amyloidogenic pathway and tau, demonstrating that the acceleration of tau hyperphosphorylation is promoted by soluble Aβ oligomers and, in turn, p-tau enhances the formation and aggregation of Aβ plaques [116].

Therefore, targeting tau phosphorylation is another important strategy in AD therapeutics, in particular the inhibition of tau protein kinases to prevent tau phosphorylation and the concomitant microtubule instability. Of the tau protein kinases, glycogen synthase kinase 3 (GSK3) has possibly aroused the most interest [117]. Specifically, valproate, lithium, tideglusib, caffeine, and several other GSK3 inhibitors have been studied in pre-clinical and clinical trials as therapeutic candidates for AD tauopathy [4]. Similarly, the reduction of tau oligomerization, prevention of microtubule stabilization, and enhancement of tau degradation are other strategies currently being studied to improve AD therapy management [4].

### Neurotransmission

The cholinergic system may be strongly affected in AD. Specifically, the degeneration of cholinergic neurons in the basal anterior brain has been reported to play a predominant role in the progression of the disease [118]. Much like tau hyperphosphorylation, cholinergic deficit is also related to Aβ. More than two decades ago, Pittel et al*.* showed the potentiation of the non-amyloidogenic pathway by activating the cholinergic receptors of the cerebral cortex and cerebellum and the consequent decrease in Aβ formation [119].

The inhibition of acetylcholinesterase (AChE) causes an increase in acetylcholine in the synaptic space and, therefore, an increase in cholinergic activity. Indeed, three of the four marketed AD drugs are inhibitors of AChE, namely Donepezil, Rivastigmine and Galantamine. They were approved by the FDA between 1998 and 2001, and since their inception have been used in mild or moderate phases of the disease [120]. However, these drugs have poor effectiveness, with patients continuing to show progressive cognitive impairment, suggesting that these compounds have only palliative effects. In contrast, their adverse effects, due to peripheral cholinergic hyperactivity, reduce patients' adherence to the treatments and therefore limit their success [120, 121].

AD neuronal pathogenesis also extends to the glutamatergic system, especially in later stages of the disease. At the central level, glutamate intervenes in most excitatory signals and participates in several physiological processes, such as neurogenesis, synaptic plasticity, memory, and learning [122]. Glutamatergic excitotoxicity is due to a massive influx of Ca^2+^ ions. In AD, this toxicity is mainly due to the overstimulation of NMDARs caused by deposits of Aβ and tau tangles. Likewise, the increase in Aβ levels induces a decrease in glutamate reuptake by glial cells, which translates into an increase in neuronal death [123]. Memantine, a non-competitive NMDAR antagonist, was approved for AD treatment in 2003 by the FDA and is used in moderate to severe phases. Like AChE inhibitors, it acts at a symptomatic level, reducing the neurotoxic effects caused by glutamatergic excitotoxicity and improving memory and learning processes. Although its effectiveness is superior to that of AChE inhibitors, it does not stop the disease progress or resolve the pathogenesis of AD [4].

In terms of neurotransmission, other routes related to the development of AD have recently come into therapeutic focus, particularly the GABAergic system, the serotonin receptor, and the modulation of histaminergic and adenosine receptors [4]. However, further research is needed to clarify the roles of these systems in AD.

### Oxidative stress

Oxidative stress crucially drives many disorders, especially those inherent to the CNS, where there is very high oxygen consumption but normally low concentrations of antioxidant enzymes [124]. In 2001, Nunomura et al*.* reported the level of oxidative stress in the early stages of AD [125]. Unexpectedly, oxidative damage decreases with disease progression and the formation of senile plaque and tau tangles, suggesting the existence of compensatory mechanisms. Some researchers have suggested that the appearance of both senile plaque and tau tangles is enhanced with increasing oxidative stress. However, other authors have claimed that the participation of Aβ in the deregulation of the intracellular homeostasis of Ca^2+^ and different mitochondrial mechanisms instead lead to the appearance of oxidative processes [124].

There is inconsistent evidence about the effect of exogenous antioxidants such as vitamins, carotenoids, phytochemicals, and synthetic compounds against already established oxidative stress damage. However, rutin, curcumin, and melatonin, potent antioxidant compounds, have been shown to possess several beneficial roles apart from their powerful antioxidant effects, such as amyloid-disaggregating properties and anti-inflammatory activity [126]. Likewise, boosting endogenous antioxidant activity is another strategy for the oxidative stress approach to AD. The nuclear factor 2 (Nrf2) /antioxidant response element (ARE) cascade is the primary endogenous antioxidant pathway. The translocation of Nrf2 from the cytosol into the nucleus is blocked in AD. Thus, drugs inducing the Nrf2/ARE pathway could be an interesting approach for the treatment of AD [4].

### Neuroinflammation

Neuroinflammation is the physiological response of the CNS immune system against molecular alterations in the brain tissue. Growing evidence suggests that the pathogenesis of AD is not restricted to neurons, but also involves immunity in the brain [66]. Thus, the activation of astrocytes and microglia, resident immune cells of the CNS, is a hallmark of neuroinflammation that is observed in most neurodegenerative conditions, including AD [127]. However, the underlying molecular mechanisms remain unclear. In general, the activation of glial cells induces many biochemical and cytological changes, such as the production of ROS, the secretion of proinflammatory cytokines, and the degradation of neuroprotective retinoids, thus endangering the surrounding healthy neurons. Similarly, external factors, such as systemic inflammation and obesity, likely interfere with immune processes in the brain and further promote disease progression [65]. Glial cells, mainly composed of astroglia, microglia, and oligodendroglia, become activated and adopt different gene expression profiles in pathological conditions, which leads to a neuroinflammatory response [128]. This response causes an overproduction of pro-inflammatory cytokines, which gives rise to an increase of amyloidosis state, looser maintenance of the myelin that surrounds CNS axons, an increase of ROS, mitochondrial damage, and ER stress, among other effects. Neuroinflammation finally contributes to the neurotoxic processes that produce neuronal death in AD [128].

Epidemiological data show the protective effect of anti-inflammatory agents in neurodegenerative diseases. Thus, the hypothesis linking neuroinflammation to the pathogenesis of AD has gained strength in recent years, even suggesting that its onset occurs long before memory impairment becomes clinically evident [129]. This has also promoted studies of NSAIDs in AD pre-clinical and clinical trials. In the context of AD NSAIDs may not only inhibit cyclooxygenase, but also target α-secretase or maintain Ca^2+^ homeostasis [4]. However, although epidemiological and observational studies have highlighted the beneficial effect of NSAIDs in reducing the symptoms and progression of AD, randomized clinical trials and meta-analyses have failed to corroborate this significantly [130].

### Mitochondria damage approach

The implications of mitochondrial dysfunction, mainly caused by ROS overproduction have also recently been described in the pathogenesis of AD [131]. Controlling ROS production is considered even more promising than traditional antioxidant strategies, which only act against accumulated ROS. Some molecules that have been studied in terms of mitochondrial anti-oxidants are coenzyme Q10, L-carnitine, triphenylphosphonium, and lipoic acid [132]. The latter also increases acetylcholine production and down-regulates the expression of pro-inflammatory cytokines [133]. Dimebon, an anti-histaminic drug, was also evaluated for its activity towards mitochondria and shown to block mitochondrial permeability transition pore opening, which translates to protection against cellular dysfunction and apoptosis processes [134]. However, the clinical translation of mitochondrial therapeutics has not yet occurred [135] and further studies are needed to elucidate the specific role of mitochondria in AD development.

### Metabolic alterations

In recent years, several studies have focused on clarifying the relationship between dementia and metabolic disorders such as diabetes, obesity, dyslipidemia, and hypertension. The alteration of the insulin pathway, a reduction in the expression of its receptors, an increase in free fatty acids and cholesterol, an increase in lipogenesis, and altered vascular function are some comorbidities related to the appearance of AD [7]. All these processes alter the clearance of Aβ in brain tissue, stimulate the hyperphosphorylation of tau, promote astrocyte and microglia activation and the secretion of ROS, and, ultimately, exacerbate the signs and symptoms of AD [136]. This highlights the importance of addressing both disorders as a single entity and the need to elucidate the underlying molecular connections to clarify the relationship between them and, therefore, their etiology. Thus, statins or antidiabetic drugs are emerging as potential, promising therapeutic candidates in AD management [137, 138].

## Controlled drug delivery systems

Until the mid-twentieth century, the most common pharmaceutical formulations were tablets, capsules, and syrups. These types of formulations meant an average of 3–4 administrations per day for patients. Likewise, many drugs with limited solubility and bioavailability had restricted use in the clinic, regardless of the therapeutic potential of the active molecule. Controlled drug delivery systems address many of these obstacles.

In 1952, Smith Klein Beecham designed the first controlled release system (CRS) called “Spansule® technology” [139]. This resulted in the first generation (1G) of CRS, which focused on the control of release kinetics. Until the 1980s, 1G CRS were most productive, with a large number of new formulations with easy administration (mainly oral and transdermal routes) [140]. The second generation (2G) focused on developing more advanced systems, such as CRS of intelligent polymers sensitive to the environment, zero-order releases, depot formulations aimed at very long administrations (months) or hydrogels. This generation was not as productive as the 1G in terms of the number of formulations that entered into clinical practice, due to the complexity of development presented by these new systems [139].

It was not until 2010 that the third generation of CRS (3G) emerged. 3G CRS comprise present and future developments to improve the clinical problems noted with 2G CRS. Efforts have focused on developing CRS with more predictable kinetics through in vitro methods, selective releases, such as longer-lasting systems and easy administration, and, overcoming the physicochemical and biological barriers that were not breached by 2G CRS [140]. Currently, the use of different nanocarriers is one of the most widely used state-of-the-art technologies to overcome the disadvantages of available drugs and offer new theragnostic approaches, especially in those diseases whose affected organs are difficult to access, such as AD.

The main properties that a device should possess to be a CRS are: (i) release the drug at a predetermined rate; (ii) can be administered locally or systemically; (iii) remain in the body for a specified period of time; (iv) target the carried drug to the specific site of action [141]. In addition, due to Fick’s law, diffusion is the mechanism that governs the controlled release of a drug and depends on the aqueous solubility, ionization, PKa, stability, partition coefficient, and molecular weight of the drug [141].

The general classification of CRS is based on the mechanisms that govern the release process and the entrapment of the drug [142]. Thus, the main types of CRS are: (i) diffusion-controlled CRS, which act as reservoirs and monolithic systems; (ii) water penetration-controlled CRS, where osmotic and swelling processes control the release of the drug; (iii) chemically-controlled CRS, which can be designed as biodegradable reservoirs and monolithic systems or biodegradable polymer backbones with pendant drugs, where the release ratio is managed by chemical reactions between the device and the medium; (iv) responsive CRS, which act as physically or chemically responsive systems, mechanical-, magnetic-, or ultrasound-responsive systems, and biochemically-responsive self-regulated systems; (v) particulate CRS, which can be designed as microparticles, liposome systems, and polymer-drug conjugates, among others [142].

The most important CRS developed for nanomedicine applications are liposomes, micelles, nanoparticles, carbon nanotubes, graphene sheets, hydrogels, dendrimers, polyelectrolyte complex, and quantum dots. Table 2 summarizes the main characteristics of all CRS developed for biomedical applications [141, 142].

**Table 2:** Different types and main characteristics of controlled drug delivery systems for biomedical applications [141, 142]

Controlled drug delivery system	Material	Size	Structure	Types	Fabrication methods	Morphology
Liposomes	Cholesterol Phospholipids	20 nm–10 µm	Lipid based spherical shaped vesicular systems	Multilamellar Single compartment Macrovesicles	Solvent evaporation Solvent dispersion Reverse phase evaporation	
Micelles	Phosphatidyl ethanolamine, Phosphatidyl choline, PEG, PVP, PEOz, PEO, PCL, PLA…	10–200 nm	Hydrophilic inner core–Hydrophobic shell architecture	Polymeric micelles Lipid micelles Supermicelles	Solvent evaporation Direct dilution Salting out Dialysis Flash nanoprecipitation	
Nanoparticles	PLGA, PEG, PCL, Chitosan, Cholesterol, Palmitate, Au, Si, Ag, Fe, Cu, Pb…	10–300 nm	Nanocapsules, nanospheres, porus speheres	Polymeric Lipid Metal Gold/Silver Magnetic Silica	Solvent evaporation Double emulsion Homogenization Salting out NaBH_4_ reduction Sol–Gel synthesis	
Dendrimers	PAMAM, PPI	20–400 nm	Macromolecules with a regularly branched tree-like structure	Simple, Chiral, Micellar, Hybrid, Metallo dendrimers	Divergent synthesis Convergent synthesis Self-assembly synthesis	
Carbon nanotubes	Carbon atoms	0.5–50 nm Ø	Rolled sheets of graphene rings built from sp2 hybridized carbon atoms	SWCNTs, MWCNTs	Laser vaporization Chemical vapor deposition Arc discharge	
Hydrogels	Fibrin, collagen, hyaluronic acid, PEG, PVA, PAAM…	–	Natural/ synthetic cross-linked polymers containing a large amount of water	Temperature-responsive hydrogels, pH-responsive hydrogels, Electric-sensitive hydrogels, Ionic strength-sensitive hydrogels	Three-dimensional printing Layer-by-Layer fabrication Microfluidic-based fabrication	
Quantum dots	CdSe, CdS, ZnS, PbS…	5–50 nm	Nanosized semiconductor particles with optical and electronic properties	Imaging QDs, Drug delivery QDs, Sensor applications QDs	Colloidal synthesis Plasma synthesis Self-assembled synthesis High temperature dual injection	
Polyelectrolyte complexes	Chitosan, PGA, SDS, CMCG, fibroin, SCMC…	20–300 nm	Polymeric matrix with oppositely charged polyelectrolytes with strong but reversible electrostatic links	Nonstoichiometric water-soluble PECs, Stoichiometric insoluble PECs, Turbid colloidal stable PECs, Complex coacervates PECs	Solution spontaneous association Simple PECs titration technique	

CMCG, carboxymethyl cashew gum; MWCNTs, Multiple-walled carbon nanotubes; PAAM, poly(acrylamide); PAMAM, poly (amido amine); PCL, Poly (ε-caprolactone); PEG, Poly (ethylene glycol); PEO, poly (propylene oxide); PEOz, poly (2-ethyl-2-oxazoline); PGA, polygalacturonic acid; PLA, poly (lactide); PLGA, poly (lactic-co-glycolic); PPI, poly(propylenemine); PVP, poly (N-vinyl pyrrolidone); SCMC, sodium carboxymethyl cellulose; SDS, sodium dextran sulfate; SWCNTs, Single-walled carbon nanotubes

## Nanotechnologic strategies for Alzheimer’s disease

### Nanocarriers as diagnostic tools in AD

One of the main problems of AD is its late diagnosis due to the delayed manifestation of first clinical symptoms compared to the onset of its molecular and cellular manifestation in the brain. When the first symptoms appear, the neuronal damage is already established and irreversible. Therefore, devices that can recognize AD biomarkers in the early stages preceding memory loss and cognitive decline have attracted much interest in clinical research. Historically, the first clinical biomarker of AD was the Aβ peptide of senile plaques, which accumulates in the gray matter of AD brains. Thus, nanodevices specific for Aβ have been designed for diagnostic purposes [143].

In this context, superparamagnetic iron oxide nanoparticles (SPIONs) used with magnetic resonance imaging (MRI) were the first attempt to use this technology for AD diagnosis. This type of nanovehicle, which could be surface modified with specific antibodies to recognize AD biomarkers, has shown elevated targeting in in vitro, in vivo, and ex vivo AD models. Thus, SPIONs coated with ganglioside carbohydrate (sialic acid), Aβ1-42 antibody, and curcumin are MRI agents used to identify Aβ plaques [144, 145]. Similarly, enhanced diagnosis was provided by liposomes modified with gadolinium and ET6-21, an amyloid-targeting ligand, compared to conventional MRI in an in vivo model of AD [146]. The preferred administration route for these kinds of diagnostic nanocarriers is intranasal since it is non-invasive, allows penetration across the BBB, and avoids systemic adverse effects. A hybrid surface-modified graphene oxide with both tau and Aβ antibodies linked to magnetic core–plasmonic coat nanomaterials was also developed. This nanodevice readily detected Aβ and tau proteins in an in vitro model of AD [143].

Another diagnostic application of nanocarriers is the specific binding of disease-specific proteins. As explained above, CFS and plasma show some of the molecular alterations that occur in the brain parenchyma of AD patients, with levels of Aβ peptides, tau, NfL, and SNAP-25, among others, increased in both. Nanocarriers could act as uptake tools to monitor these biomarkers, thus contributing to a solid diagnosis of the stage and severity of AD pathogenesis [143].

### Nanocarriers as therapeutic tools in AD

The complexity of the therapeutic approach to AD lies not only in its unknown etiology and the lack of available effective treatments, but also in the restricted access to the affected organ. Three biological barriers control the passage of most substances into the brain and thus significantly limit drug access: the BBB, blood-CSF barrier (BCSFB), and ependymal barrier [143]. For orally administered drugs, the gastrointestinal barrier and hepatic first-pass effect must also be added [147]. Likewise, clearance mechanisms and efflux pumps significantly reduce the half-life of drugs in the body, thereby contributing to the reduction of pharmacological effectiveness [148].

Apart from accessibility problems, drugs administered to the CNS, as well as other organs, must fulfill specific biopharmaceutical characteristics that confer high bioavailability, but these are not always present. Although their pharmacological activity might be relevant, many drugs have physicochemical disadvantages, such as low solubility, short stability, and high molecular weight, that significantly reduce their bioavailability and, therefore, their final therapeutic effectiveness [149]. Additional challenges that CNS therapies face include the presence of peripheral adverse effects and the difficulty of finding the therapeutic threshold and maintaining it over time.

To overcome all these obstacles, the therapeutic potential of drug-loaded nanocarriers in different neurodegenerative diseases has been explored [150]. The high surface-to-volume ratio of nanocarriers and the possibility of surface functionalization with desired ligands are the two main exploited characteristics of these devices for drug delivery to CNS [150]. In addition, Aβ targeting has been the main objective of nanomedicine to date. Three methods from nanotechnology have been used to target and modify senile plaques: (i) genetic regulation and/or inhibition of the synthesis of Aβ peptide; (ii) inhibition and/or delay of the Aβ nucleation dependent process; (iii) clearance of already-formed Aβ plaques [143]. Álvarez-Erviti et al*.* employed naturally inert exosome nanocarriers to deliver BACE1-siRNA into the brain and silence BACE expression, thus inhibiting the cleavage of APP and, in turn, the overproduction of Aβ peptide [151]. Recent findings related to the most common nanovehicles in preclinical assays of AD are described in depth below.

## Lipid-based nanoparticles for Alzheimer’s disease

Lipid-based NPs (LNPs) are among the most used controlled drug delivery systems to target and deliver drugs to the brain. Their safety, biocompatibility, and biodegradability make these systems an interesting choice for drug nanocarriers. Furthermore, since their inherent lipidic structure is similar to the lipid composition of the BBB, their penetration into the brain by the transcellular pathway is favored [152]. The core of LPNs is commonly composed of monoglycerides, triglycerides, fatty acids, fatty alcohols, mixtures and waxes [153]. The most common LNPs are liposomes, solid lipid NPs (SLN), and nanostructured lipid carriers (NLCs), which mainly differ in the disposition of the lipid layers, morphology, loading capacity, average particle size, and electrokinetic behavior [154]. This type of nanocarrier can encapsulate both hydrophilic and hydrophobic drugs, which significantly increases their versatility [155]. However, their low stability, complex fabrication method, unexpected polymorphic transitions, and drug release during storage are some disadvantages that complicate large-scale production and, therefore, widespread use [154]. However, many efforts are being made to improve these issues and the potential of LNPs as diagnostic and therapeutic tools in AD and other CNS diseases [156]. Recent preclinical studies have produced interesting results by combining state-of-the-art LNPs and AD animal models (Table 3).

**Table 3:** Selected relevant pre-clinical studies based on recent LNPs for the diagnosis and treatment of AD

Loaded molecule	Lipid matrix	Surface modifications	Dose	Admin. route	In vitro/In vivo model	Results	Refs
PE	Lecithin Stearic acid Cholesterol	–	50 mg/kg	v.o	AlCl 3-induced Wistar rats	PE shows a protective effect in the AD animal model NPs improve the PE effectiveness in novel object recognition test, increase of biomarkers of antioxidant activity, lipid peroxidation reduction, and neurofibrillary tangles and the senile plaques reduction	Almuhayawi et al. [57]
α-bisabolol	Cholesterol	–	5–10 μg/ml	incubation	Aβ25–35 Neuro-2a cells	NPs significantly suppress the production of free radicals, reduce β-secretase, caspase-3, cholinesterase activities and expression of Bax, and induce expression of Bcl-2 proteins	Sathya et al. [164]
Quercetin	Cetyl palmitate miglyol-812	Transferrin	10–30 µM	Incubation	hCMEC/D3 cells	Quercetin-NPs are not toxic for hCMEC/D3 cells and permeate more the BBB than free drug. Moreover, NPs inhibit fibril formation of Aβ peptide	Pinheiro et al. [161]
EPO	GMS	–	1250–2500 IU/Kg	i.p	Aβ42-induced Wistar rats	EPO-NPs significantly restore memory in treated rats compared to those treated with free EPO EPO-NPs reduce the oxidative stress, ADP/ATP ratio, and Aβ plaque deposition in the hippocampus more effectively than the free EPO	Dara et al. [165]
Curcumin	Caprylic/capric triglycerides, sorbitan monostearate	PCL	1–10 mg/kg	i.c.v	Aβ42-induced Swiss Albino mice	Curcumin-NPs display significant neuroprotection against Aβ42-induced behavioral and neurochemical changes in the AD-induced mice model	Giacomedi et al. [158]
α-bisabolol	Cholesterol	–	5–10 μg/ml	Incubation	Aβ25–35 Neuro-2a cells	α-bisabolol-NPs protect the Neuro-2a cells from Aβ induced neurotoxicity and inhibited Aβ aggregation	Sathya et al. [163]

EPO, Erythropoietin; GMS, Glycerin monostearate; hCMEC/D3 cells, Immortalized human cerebral microvascular endothelial cells; PCL, poly(ɛ-caprolactone); PE, pomegranate extract

Almuhayawi et al*.* designed pomegranate extract-loaded LNPs to evaluate their effects in an aluminum chloride-induced rat model of AD [157]. Pomegranate extract is highly enriched in alkaloids and tannins, which have powerful antioxidant effects. The results showed that, compared to untreated controls, animals treated with such LNPs exhibited decreased NFTs and Aβ deposits, improved cognitive test scores, and increased biomarkers of antioxidant activity in the brain homogenates. Similarly, Giacomeli et al*.* investigated the neuroprotective effects of curcumin-loaded LNPs in a mice model of AD [158]. The therapeutic potential of curcumin has been widely recognized for many diseases, but its low solubility and, consequently, reduced bioavailability limit its pharmacological effect [159]. In this study, the authors developed a hybrid nanocarrier of a lipidic core with a PLC coat to confer better solubility. Animals treated with this nanocarrier showed improved spatial memory and reduced neuroinflammation biomarker levels in serum and the hippocampus and cortex homogenates. Pinheiro et al*.* combined natural products and LNPs. The bioactive load was quercetin, a flavonoid present in many vegetables and fruits with strong antioxidant activity. The authors went one step further and coated the LNPs with transferrin to enhance transport across the BBB [160]. These LNPs were found to be non-toxic for hCMEC/D3 cells, a frequently used in vitro model of human BBB, and to enhance the penetration of quercetin through the BBB. Moreover, these LNPs inhibited Aβ1-42 fibril formation in an in vitro model [161]. Similarly, α-bisabolol, a sesquiterpene alcohol found in *Matricaria chamomilla* essential oil with demonstrated anti-plasmodial, anti-microbial, anti-inflammatory, anti-cancer and anti-cholinesterase properties, has significantly reduced bioavailability due to low solubility [162]. Sathya et al*.* developed α-bisabolol-loaded Cholesterol LNPs and explored their neuroprotective effects in an Aβ-induced in vitro model of Neuro-2a cells, a fast growing mouse neuroblastoma cell line [163, 164]. These studies show that such LNPs possess antioxidant potential, significantly reduce β-secretase, caspase-3, and cholinesterase activities, inhibit Aβ aggregation, and protect Neuro-2a cells from Aβ-induced neurotoxicity by reducing the expression of Bax and inducing the expression of Bcl-2 proteins [163, 164].

Finally, Dara et al*.* recently investigated the therapeutic potential of erythropoietin (EPO) in AD. EPO is neuroprotective in several diseases, such as spinal cord injury, cerebral ischemia, epilepsy, and diabetic neuropathy [165]. Moreover, EPO contributes to neuronal survival and the regulation of neurogenesis in both Parkinson’s disease and AD [166, 167]. However, EPO has restricted penetration through the BBB due to its hydrophilicity, rapid clearance from the bloodstream, and high molecular weight. Encapsulated in SLNs, EPO reduced oxidative stress and Aβ deposition in the hippocampus more efficiently in an Aβ42-induced Wistar rat model of AD [165]. Moreover, animals treated with EPO-LNPs exhibited increased spatial memory compared to free EPO-treated littermates.

## Polymeric-based nanoparticles for Alzheimer’s disease

Due to their versatility and ease of fabrication by different methods, polymeric NPs (PNPs) are among the most used carriers in nanomedicine applications [168, 169]. Their mean average size is between 10 and 1000 nm and they can load high amounts of both hydrosoluble and hydrophobic drugs [170]. Depending on the polymer composition, which can be natural or synthetic, PNPs can have both positive and negative surface charges. This characteristic significantly conditions their biological behavior, muco-adhesiveness, and penetration [171].

PNPs can be formulated as nanocapsules and nanospheres. Nanocapsules possess a vesicular structure in which drugs are dissolved in a liquid core surrounded by the polymeric capsule. In contrast, nanospheres are composed of a polymeric matrix in which drugs are dispersed in the matrix gaps or adsorbed onto the sphere surface [172]. The polymers most used to manufacture PNPs are polylactide (PLA), poly(lactide-co-glycolide) (PLGA), chitosan, polyethyleneimine (PEI), and poly-ε-caprolactone (PCL), all of which were approved by the FDA for biomedical applications twenty years ago [173]. These kinds of vehicles have many important advantages, such as their high loading capacity, controlled drug release kinetics, surface modifications for brain targeting, safety, biodegradability, biocompatibility, and easy elimination [168]. However, these systems also have some disadvantages that limit their use, especially the need for organic solvents during their fabrication. Nevertheless, their pharmacological potential has encouraged many researchers to explore the potential of PNPs for the diagnosis and therapeutic management of different neurodegenerative diseases [150]. Table 4 summarizes recent advances on the preclinical evaluation of state-of-the-art PNPs in AD.

**Table 4:** Selected relevant pre-clinical studies based on recent PNPs for the diagnosis and treatment of AD

Loaded molecule	Polymeric matrix	Surface modifications	Dose	Admin. route	In vitro/in vivo model	Results	Refs
Lutein	PLGA	Chitosan	0–20 mM	Incubation	SHSY-5Y cells RPMI 2650 cells	NPs are highly deposited in brain following i.n. route and demonstrated to possess significant ROS scavenging activity	Dhas et al. [174]
			4 mg LT/kg	i.n	Sprague Dawley rats		
NAP	PLA	TPL PEG	10 μM	Incubation	bEnd.3 cells PC12 cells HT22 cells CTX-TNA2 cells	TPL-PNPs show higher binding affinity to either GT1b ganglioside receptor or brain capillary endothelial bEnd.3 cells, increase the BBB-penetration and neuron-targeting efficacy, enhance ROS scavenging ability and protect microtubule from Aβ25‐35-induced neurotoxicity, inhibit okadaic acid-induced tau aggregation and neuronal apoptosis, improve the cognitive performance of treated mice, down-regulate the tau phosphorylation level, promote axonal transport and attenuate microgliosis	Guo et al. [185]
			6–24 μg NPs/kg/day	N.A	ICR mice		
CDs GMP	Chitosan	Eu(NO3)3 CuCl2	0.67 mg/ml	NPs-CSF sample incubation	AD rats	Developed NPs act as a ratiometric fluorescent probe for the detection of Aβ monomers. In CSF and various brain tissues of rats, developed NPs are able to recognize the Aβ peptide and fluoresce, thus leading to its detection and quantification	Liu et al. [181]
Phytol	PLGA	–	5–10 µg/ml	Incubation	Neuro-2a cells	PNPs increase the lifespan, chemotaxis behaviour and decrease Aβ deposition and ROS production in the in vivo models of AD. Moreover, PNPs treatment downregulate the expression of AD associated genes viz Aβ, *ace*-1 and *hsp*-4 upregulate the gene *dnj*-14, involved in the longevity of nematodes, and reduce the expression of Aβ peptide at the protein level	Sathya et al. [176]
			25, 50 and 100 μg/ml	Exposition	*Caenorhabditis elegans* (CL2006, CL4176)		
Curcumin	PLGA	[Gd]DTPA Chitosan IgG4.1 K16ApoE	100 µCi/100 µL	i.v	Tg2576 mice	NPs improve BBB transcytosis by coating with a K16ApoE NPs enhance MRI contrast to detect Aβ plaques	Ahlschwede et al. [180]
EGCG/AA	PLGA	PEG	15–500 μg/ml	Incubation	BMVECs	NPs effectively penetrate through the in vitro BBB without damaging the BBB integrity. NPs treatment reduce neuroinflammation, Aβ plaque burden, soluble and insoluble Aβ42 peptide levels and enhance synapsis expression, spatial learning and memory processes	Cano et al. [178]
			40 mg/kg/day	v.o	APP/PS1 mice		
DBP	PLGA	–	2.5 mg/kg	i.v	5XFAD mice	Inhibition Aβ aggregation in vitro. Attenuation of Aβ accumulation, neuroinflammation, neuronal loss and cognitive dysfunction	Jeon et al. [182]

AA, ascorbic acid; BMVECs, brain microvascular endothelial cells; CDs, carbon dots; CSF, cerebrospinal fluid; DBP, Vitamin D-binding protein; EGCG; Epigallocatechin-3-gallate; GMP, guanosine monophosphate disodium; NAP, neuroprotective peptide; ROS, Reactive oxygen species; TPL, fusion peptide comprising a BBB-penetrating peptide TGN and a neuron binding peptide Tet1

The lack of effective treatments for AD has led to an incessant search for new pharmacological alternatives. Among these alternatives, natural compounds are emerging as potential therapeutic drugs for several diseases, such as cancer, diabetes, and cardiovascular and neurodegenerative diseases, including AD. Thus, Dhas et al*.* developed cationic biopolymer core/shell NPs loaded with lutein (LT), a natural dietary carotenoid mainly obtained from food such as green vegetables, eggs, and corn [174]. Although this molecule has shown promising beneficial effects, such as anti-inflammatory, anti-oxidant, and anti-cancer activity, its reduced solubility and bioavailability have restricted its use in the food and pharmaceutical industries [175]. In this study, the authors demonstrated that the developed nanocarrier possesses the optimal physicochemical characteristics for suitable in vivo administration. The PNPs have good entrapment efficiency, sustained LT release, protection of LT integrity, biocompatibility with brain cellular models, and efficient passage through an in vitro BBB [174]. Moreover, after intranasal administration in rats, LT-loaded PNPs accumulated in the brain and exhibited reduced toxicity and significant ROS scavenging activity [174]. Similarly, Sathya et al*.* evaluated the therapeutic potential of phytol-loaded PNPs in the regulation of the expression of AD-related genes and neuronal degeneration in both in vitro and in vivo models of AD [176]. As with LT, phytol is a natural compound with several pharmacological properties but poor solubility and low absorption, resulting in reduced bioavailability that limits its clinical use [177]. In the in vitro Neuro-2a cell model, phytol-loaded PNPs inhibit apoptosis-mediated cell death and cholinesterase activity. In a transgenic nematode AD model, these PNPs were found to increase chemotaxis and lifespan and reduce ROS production and Aβ deposition. Furthermore, PNP treatment upregulated a gene involved in the longevity of nematodes, downregulated the expression of several AD-associated genes, and reduced the expression of Aβ peptide at the protein level [176].

Cano et al. also studied natural-compound-loaded PNPs for AD [178]. In this study, the selected molecule was EGCG (see also above), which has shown therapeutic activity in many diseases, such aas breast cancer, diabetes mellitus, Down syndrome, and different neurodegenerative diseases, but its instability in water solutions and in vivo administrations reduce its effectiveness [179]. Co-encapsulating EGCG into PEGylated PLGA NPs with ascorbic acid (AA) to prevent auto-oxidation produces a nanosystem with significantly enhanced EGCG integrity, which is correlated with improved EGCG effectiveness in a variety of biological assays relevant to AD [178]. EGCG/AA PNPs readily cross the BBB both in vitro and in vivo. Moreover, compared to free EGCG, EGCG/AA NPs exhibit improved bioavailability and pharmacokinetic profile and, in an AD mouse model, improved memory learning processes, reduced cognitive decline, neuroinflammation, and Aβ plaque burden, and increased synaptic expression.

Taken together, these findings highlight the relevance of natural compounds as promising therapeutic strategies in AD management. However, PNPs loaded with natural compounds have not only been studied as therapeutics, but also for diagnostic purposes. In this regard, Ahlschwede et al*.* developed curcumin-loaded PLGA NPs functionalized with K16ApoE, a BBB penetration peptide. The authors aimed to create an effective tool for detecting cerebrovascular Aβ and treating cerebral amyloid angiopathy, which is widely observed in AD development [180]. Using a quartz crystal microbalance with dissipation monitoring technology, the authors found that developed PNPs effectively migrated from the blood flow to the vascular endothelium. As expected, K16ApoE coating significantly improved BBB transcytosis and provided specific MRI contrast to detect brain Aβ plaques. Moreover, K16ApoE-PNPs also showed specific targeting of vasculotropic DutchAβ40 peptide that accumulated in the cerebral vasculature. Despite this study’s innovations, in vitro and in vivo assays are still required to describe the therapeutic effects of curcumin-loaded K16ApoE-PNPs on memory impairment, cognitive decline, and molecular alterations related to AD development. Liu et al*.* used nanotechnology for diagnosis. The authors designed carbon dots (CDs) sensitized lanthanide infinite coordination polymer (ICP) NPs and a PNPs-based ratiometric fluorescent probe mainly composed of CDs, Europium Nitrate (Eu(NO_3_)_3_), and Cu^2+^ [181]. The detection technique was based on the competitive coordination interaction of Cu^2+^ between the guest CDs and Aβ monomer. Briefly, in the absence of Aβ, the coordination interaction between CDs and Cu^2+^ disrupts the antenna effect, leading to the fluorescence quenching of Eu^3+^. When developed PNPs contact an Aβ-enriched sample, the stronger coordination between Aβ monomer and Cu^2+^ restores the red fluorescence of Eu^3+^, leading to the detection and quantification of Aβ peptide by fluorescence. In this study, developed PNPs were exposed to CSF/brain tissue samples of AD rats. The authors showed that their method is highly sensitive for the in vivo analysis of Aβ monomer, thereby demonstrating the utility of this ratiometric fluorescent probe.

Some endogenous substances have been also described as pharmacological tools for AD. In that sense, Jeon et al*.* explored the therapeutic potential of Vitamin D-binding protein (DBP) [182]. DBP is a glycoprotein that is highly expressed in a wide variety of cells and tissues and has been shown to play important roles in several physiological processes. Likewise, several studies have highlighted that DBP levels are altered in the serum and CSF of AD patients and possess optimal properties for Aβ binding, and thus peripheral clearance [183, 184]. However, previous studies have shown that plasma DBP has a relatively short half-life. Jeon et al*.* aimed to encapsulate DBP into PLGA matrices to prolong the presence of DBP in the bloodstream and evaluate their effectiveness in an Aβ-overexpressing mice model of AD. The developed nanocarrier inhibited the polymerization and accumulation of Aβ in both in vitro and in vivo models, ameliorated Aβ-associated neuroinflammation and neuronal loss, and significantly reduced the cognitive impairment of treated transgenic mice.

Finally, Guo et al*.* recently developed a PNP coated with both a BBB-penetrating ligand and a neuron-targeting ligand for carrying the neuroprotective peptide NAP. NAP has been shown to provide neuroprotective activity against NMDA receptors and Aβ-mediated excitotoxicity by interacting with glial and neuronal tubulin, thereby promoting microtubule assembly and the protection of the neuronal cytoskeleton [185]. As explained above, the clinical application of NAP is restricted due to its enzymatic degradation, ineffective neuron targeting and reduced half-life in the bloodstream [185]. The encapsulation of NAP in the nanocarrier improved its stability in vivo. Likewise, coating the PNP’s surface effectively increased the accumulation of developed NPs in brain neurons. The evaluation of its pharmacological properties highlighted that NAP-PNPs promote the alleviation of oxidative stress, microtubule disruption, the attenuation of neuroinflammation, and the inhibition of tau aggregation and apoptosis processes. Likewise, a rescue of the memory deficits and spatial learning in AD mice was observed after PNP treatment. Moreover, treatment with these PNPs also prevented tau hyper-phosphorylation and restored axonal transport.

## Metal-based nanoparticles for Alzheimer’s disease

Metal-based NPs (MNPs) are the most relevant inorganic nanocarriers because of their widespread use in nanomedicine applications [186]. These vehicles usually have an average size of 10 to 100 nm. Moreover, their high surface area allows for coating with different molecules (e.g., antibodies, genes, peptides), which lets them act as biosensors and targeting tools. MNPs are commonly fabricated with gold, silver, iron, zinc, and copper, all of which provide different properties to the final nanocarrier [187]. MNPs have demonstrated anti-microbial, anti-cancer, anti-inflammatory, and anti-oxidant properties, among others [188].

Different methods are employed in the fabrication of MNPs, with top-down methods (e.g., mechanical milling, laser ablation, and ion sputtering) and bottom-up methods (e.g., solid-state methods, liquid-state synthesis methods, gas-phase methods, and electrochemical deposition). Note that the selected fabrication method significantly conditions the final physicochemical properties, morphology, and long-term stability of MNPs [189]. Green synthesis methods, an emerging trend of nanotechnology, can overcome the toxicity problems, high cost, and reaction complications of conventional fabrication methods, so they have attracted much recent interest. Green chemistry incorporates novel techniques to reduce the health and environmental burdens of conventional techniques. Some of the most used green synthesis methods involve biological methods with different microorganisms and their enzymes and using plant extracts. The main advantages of these novel techniques are reduced costs, ease of scaling up for large-scale production, and the complete elimination of energy, high pressure and temperature, and toxic chemicals [190].

Much current research focuses on MNPs because of their multiple benefits and versatility for use in disease diagnosis and treatment, labeling optoelectronic recorded media, cosmetics, and sensor technology, among others [189]. In addition, the magnetic properties of some of these MNPs can be exploited to enhance the accumulation of these NPs in specific organs since they can be detected and manipulated by remote magnetic fields [191]. Table 5 displays relevant studies involving the latest smart MNPs and AD preclinical models.

**Table 5:** Selected relevant pre-clinical studies based on recent MNPs for the diagnosis and treatment of AD

Loaded molecule	Metal core	Surface modifications	Dose	Admin. route	In vitro/in vivo Model	Results	Refs
–	Iron oxide	NIR Fluorescent probe	10 ng/mL	Incubation	Aβ42 induced SH-SY5Y cells	MNPs can simultaneously perform in vivo NIR fluorescence and magnetic resonance imaging of Aβ plaques in the brain of transgenic mice, prevent Aβ aggregation, disaggregate preformed Aβ fibrils and play a protective effect on the toxicity of human neuroblastoma cells induced by Aβ42	Cai et al. [201]
			0.2 mmol Fe/kg	i.v	APP/PS1 mice		
–	Iron oxide	chitosan	NA	Exposition	Synthetic urine	MNPs were used to fabricate a highly sensitive AChE electrochemical biosensor. The novel biosensor exhibits the lower limit of detection of ACh that has been reported in the literature. It also shows good recoveries in the determination of ACh in synthetic urine	da Silva et al. [192]
–	Selenium	Chondroitin sulfate	1.45 µg/mL	Incubation	SH-SY5Y cells	MNPs inhibit Aβ aggregation, protect SH-SY5Y cells from Aβ42-induced cytotoxicity, decrease okadaic acid-induced actin cytoskeleton instability, In addition, decrease the levels of ROS and MDA, increase the levels of GSH-Px, and attenuate the hyperphosphorylation of tau by regulating the expression of GSK-3b	Gao et al. [195]
–	Cerium dioxide	Zirconium	4 mg/m^3^ for 3–5 h/day	i.n	5xFAD mice ApoE-/- mice	The inhalation exposure to the MNPs promotes changes in forced motor performance and exploratory motor activity in ApoE-/- and 5xFAD mice, respectively	Wahle et al. 2020
Quercetin	Gold / Palladium	Polysorbate 80	5–40 µg/mL	Incubation	SH-SY5Y cells	MNPs activate autophagy of SH-SY5Y cells, promote the fusion of autophagosomes and lysosomes, accelerate the clearance of Aβ, and protect SH-SY5Y cells from Aβ -induced cytotoxicity damage. MNPs are not toxic both in vitro and in vivo	Liu et al. [194]
			1 mg/kg	i.v	Nude mice		
–	Iron oxide Cadmium sulfide	*F. oxysporum* and *Verticillium sp.* proteins	5 − 100 μg/mL	incubation	Neuro2a cells	MNPs do not affect the viability of neuroblastoma cells and show dual properties of inhibition and disaggregation of Tau fibrillar aggregates	Sonawane et al. [198]
–	Palladium	Bioactive Hydrogen	6.25–25 µg/ml	incubation	N2a-SW cells	Developed MNPs are able to recover mitochondrial dysfunction, inhibit Aβ generation and aggregation, block synaptic and neuronal apoptosis and promote neuronal energy metabolism by eliminating oxidative stress and activating the anti-oxidative pathway, consequently ameliorating the cognitive impairment in AD mice	Zhang et al. [200]
			2µL of MNPs (0.5–2 mg/ml)	Stereotaxic injection	APP/PS1 mice		
–	Silver	PrP_95-110_	1.2 nM	Incubation	Aβ enriched CSF and serum	Authors report an electrochemical method for the detection of Aβ oligomer with a peptide as the bio-receptor and silver MNPs aggregates as the redox reporters	Xing et al. [193]
CQ	Gold	–	0.5 mg/mL	Incubation	PC12 cells	In this system, CQ is released only upon exposure to conditions in which H_2_O_2_ levels are high, such as those in Aβ plaques. NPs inhibit Aβ40 aggregation, and reduce the cell membrane disruption, microtubular defects and ROS-mediated apoptosis	Yang et al. [199]
			5, 10, 20 mg/kg	i.v	ICR mice		

Ach, acetylcholine; CQ, metal chelator Clioquinol; CSF, cerebrospinal fluid; GSH-Px, glutathione peroxidase; MDA, malondialdehyde; NIR, phenothiazine-based near-infrared fluorescent dye; PrP_95-110_, cellular prion protein; ROS, reactive oxygen species; STZ, streptozotocin

Currently, using MNPs for diagnosis purposes is being widely studied. For example, da Silva et al*.* recently developed a highly sensitive AChE electrochemical biosensor based on an electrode modified with iron oxide NPs and a deep eutectic solvent [192]. Enzyme-modified electrode sensors offer a powerful tool for real-time diagnostics that overcomes the drawbacks of traditional analytical approaches, such as time-consuming processes, high cost for measurements, and the need for highly specialized staff [192]. Likewise, their capability to promote the fast electron-transfer kinetics of iron oxide NPs means they had optimal properties for use in the developed biosensor. Da Silva et al*.* immobilized AChE on a polymeric-based film pre-formed in modified electrodes, which was composed of iron oxide NPs and an acid eutectic dissolvent. They tested different acids to evaluate their influence on the rate of growth and electrochemical properties of the polymer films and finally selected HNO_3_. The authors used chronoamperometry to investigate the catalytic activity of the developed biosensor for ACh detection. After exposing the biosensor to synthetic urine containing known concentrations of ACh, the authors recorded the typical current–time voltage curves and demonstrated that this MNPs-based biosensor has high sensitivity, excellent reproducibility, and long-term stability, with significant recovery ratios. In a related study, Xing et al*.* investigated the development of an electrochemical technique for detecting Aβ based on silver NPs aggregates as the redox reporters and a cellular prion protein (PrP_95-110_) as the bio-receptor [193]. The fundamentals of their technique are based on the oscillation of the electrochemical signal promoted by the interaction of Aβ with the PrP_95-110_-MNPs. The specific binding of the Aβ oligomers to the PrP peptide blocks the aggregation of the MNPs’ main electro-transmitter, which decreases the electrochemical signal that can be detectable and quantifiable. The exposure of this nanodevice to both Aβ-enriched artificial CSF and the human serum of AD patients demonstrated that the developed detection method exhibits high sensitivity and specificity and requires simple manipulation, less time expense, lower expenses, and lower detection limits than those achieved by previously reported methods.

The use of MNPs as therapeutic tools for AD is also important. For example, Liu et al*.* explored the therapeutic potential of quercetin-modified gold–palladium NPs to promote the clearance of intracellular Aβ by autophagy induction and, consequently, reduce Aβ-induced neurotoxicity [194]. As explained above, quercetin is a potent natural antioxidant with limited ability to cross the BBB and easy elimination. Recent studies have shown that quercetin can accelerate the elimination of abnormal Aβ by enhancing the autophagy effect of brain cells [194]. Liu et al*.* reported that the developed MNPs are not toxic in both in vitro and in vivo and effectively cross the in vitro BBB. Furthermore, they increased intracellular autophagy levels, promoted the degradation of autophagosomes, enhanced Aβ clearance in brain cell cultures, and reduced Aβ-induced cytotoxicity. Similarly, Gao et al*.* recently evaluated multi-targeted chondroitin sulfate /selenium MNPs and evaluated their protective effect against Aβ-induced neurotoxicity [195]. Chondroitin sulfate is a glycosaminoglycan with several biological functions, such as antioxidation, anti-inflammation, and neuroprotection. Moreover, chondroitin sulfate is involved in cell migration, neurogenesis, axon growth, synaptic plasticity, neuron regeneration, the inhibition of Aβ fibril formation, and the blocking of Aβ-induced cell apoptosis [196]. Also, selenium is a potent antioxidant that plays important roles in detoxification, the protection of the immune system, and the regulation of cellular redox homeostasis. Furthermore, it also inhibits the formation of Aβ plaques and degrades preformed Aβ fibers [197]. In this new study [195], Gao et al*.* obtained promising results. Their MNPs could reduce damage to the cytoskeleton and neuronal cells and oxidative stress, inhibit the aggregation of Aβ and protect cells from its neurotoxicity, and attenuate the hyperphosphorylation of tau protein. Sonawane et al*.* also explored the effect of MNPs in Tau-related pathogenesis. The authors biologically synthesized two different protein-capped MNPs, (i) iron oxide NPs and (ii) cadmium sulfide NPs, and evaluated their effectiveness in an in vitro model of AD [198]. The surface modification of these MNPs with proteins was performed to inhibit Tau protein aggregation. To complete the biological synthesis of these carriers, two fungal species were used, *Fusarium oxysporum* and *Verticillium sp*. The composition and synthesis of both NPs were different: iron oxide NPs were synthesized extracellularly by the fungal species with transient ferromagnetic properties and capped with hydrolytic proteins. In contrast, cadmium sulfide NPs were synthesized from the extracellular sulfate-reducing enzymes secreted by the fungus when provided with mixture of salts, so the surface proteins were composed of a mixture of four different proteins that probably belonged to the group of these sulfate-reducing enzymes. Both nanocarriers did not affect the viability of cultured cells. Furthermore, both MNPs efficiently inhibited Tau aggregation, and cadmium sulfide NPs exhibited a significant dissembling of Tau NFTs.

As described above, oxidative stress is one of the most important molecular hallmarks in AD development. In AD pathogenesis, the exacerbated formation of H_2_O_2_ is favored by generalized brain oxidative stress. Metal chelators can block the detrimental effects of ROS, but their non-specific interactions with metal ions of normal cellular processes and their BBB-reduced permeability significantly limit their therapeutic success [199]. Yang et al*.* designed an H_2_O_2_-sensitive detection system composed of Clioquinol-doped gold NPs-capped mesoporous silica [199], and revealed that the conjugation of Clioquinol-MNPs on the surface of mesoporous silica leads to selective and sustained Clioquinol release under an increased H_2_O_2_ environment (e.g., surrounding of Aβ plaques). Furthermore, their nanodevice efficiently crossed the BBB, decreased Aβ self-assembly, and reduced microtubular defects, cell membrane disruption, and ROS-mediated apoptosis induced by Aβ40.

Hydrogen (H_2_) has been shown to selectively scavenge highly cytotoxic ROS. Furthermore, Hydrogen’s bio-safety and bio-diffusibility position it ahead of many compounds in AD drug discovery. However, the high solubility of H_2_ and traditional hydrogen administration routes, such as the oral intake of hydrogen-rich water, inhalation of hydrogen gas, or injection of hydrogen-rich saline, do not ensure its accumulation in the brain for the time necessary to exert its activity [200]. Zhang et al*.* developed reactive hydrogen-doped palladium NPs and evaluated their effects against oxidative stress-induced mitochondrial dysfunction in both in vitro and in vivo models of AD [200]. This was the first time that a nanosystem was shown to be able to perform a self-catalysis of carried H_2_ and realize an in situ sustained release of bio-reductive hydrogen. This innovative system effectively stores and releases bio-reductive hydrogen to selectively scavenge^**.**^OH in AD cells, inhibit Aβ generation and aggregation, reverse synaptic deficits and neuronal death, and ameliorate mitochondrial dysfunction and cognitive impairment in transgenic AD mice.

Going a step further, Cai et al*.* recently developed ultrasmall superparamagnetic iron oxide NPs (USPIONs) coupled to a phenothiazine-based near-infrared (NIR) fluorescent dye for both the diagnosis and treatment of AD [201]. This compound was shown to effectively inhibit the self-aggregation of Aβ and disaggregate preformed Aβ fibrils, as well as exhibit high fluorescence enhancement upon binding to aggregate Aβ proteins [201]. Thanks to their small size, amyloidogenic proteins, such as Aβ peptides, tend to be absorbed on the surface of USPIONs, thereby reducing circulating Aβ. Likewise, brain cells have been shown to be highly sensitive to USPIONs and their responsiveness to an external magnetic field is an important advantage to promote brain cell uptake. Moreover, the moderate heat emitted from USPIONs under the action of a low radiofrequency field represents a significant advantage since it increases the BBB’s permeability without disturbing its integrity [201]. Cai et al*.* revealed that developed USPIONs can prevent Aβ aggregation, disaggregate preformed Aβ fibrils, simultaneously perform in vivo NIR fluorescence and MRI of Aβ plaques in the brain, and perform a neuroprotective effect against Aβ42-induced toxicity.

Importantly, not only is the theragnostic potential of MNPs being studied, but also their potential neurotoxic effects. Increasing evidence shows the relevance of toxins in AD development, such as ultrafine air pollution particles. Therefore, and since nanomedicine has emerged as a promising alternative for AD management, concerns have been raised about the potential neurotoxic effects of different nanocarriers, especially MNPs. Their small size and easy penetration into human tissues by different access routes allow their high accumulation in the brain. Cerium dioxide NPs have gained much interest in recent years because of their radical-scavenging properties. For these reasons, Wahle et al*.* recently evaluated the neurotoxic effects of zirconium-doped cerium dioxide NPs in two different mice models of AD [202]. The continuous inhalation of these nanocarriers promotes changes in exploratory motor activity and forced motor performance in AD mice and increases GFAP expression in healthy mice. Thus, although MNPs represent a promising therapeutic alternative, these results highlight the importance of exhaustive research into their neurotoxicity to ensure therapeutic safety.

## Conclusions

Dementias, including AD, are the fifth-leading cause of death worldwide, accounting for 2.4 million deaths yearly. The expected increase in the number of cases of dementia in the next few decades is even more important, given that there are currently no effective disease-modifying treatments for AD [1]. In addition, even when clinical trials are initiated, many of them fail to discover a new disease-modifying drug to market: of every 100 molecules in clinical AD trials, only one reaches the market, compared to the pharmaceutical industry average ratio of 14.6:1 [1]. Without novel promising treatments in sight, increasing numbers of cases will pose an undue burden on patients who have dementia, their caregivers, and healthcare systems in general. The latest drug development pipeline shows that, in 2020, there were 121 compounds in clinical trials for the treatment of AD [10]. The lack of success in AD drug development reveals the complexity of this disease and the current challenges of AD neuropharmacology research. Suggestions to abandon the amyloid hypothesis are increasing and emerging interest is focused on combination therapies [10]. Progress in AD management depends on innovation, the assessment of new candidates, and the implementation of new trial approaches. As in other chronic diseases, such as cardiovascular disease or cancer, a learning phase preceded periods of sequential incremental success leading to meaningful treatments. Therefore, the increase of studies of combinations of new biomarkers and drug targets, combined with novel research in state-of-the-art nanocarriers, will pave the future for the more efficient delivery of bioactive molecules. Furthermore, the clinical development of biomarkers of AD progression to create treatments that are more efficacious will be one of the most ambitious challenges for future clinical practice. In that regard, recent advances on nanomedicine-drug development and novel diagnostic biomarkers could represent a promising alternative in the management of AD, as well as other neurodegenerative diseases.

## Data Availability

Not applicable.

## Abbreviations

AA: Ascorbic acid
Aβ: Amyloid-β
AD: Alzheimer’s disease
APP: Amyloid-precursor protein
BBB: Blood–brain barrier
CDs: Carbon dots
CSF: Cerebrospinal fluid
CU: Cognitively unimpaired
DALYs: Disability-adjusted life years
DBP: Vitamin D-binding protein
EGCG: Epigallocatechin-3-gallate
EOAD: Early onset AD
EPO: Erythropoietin
FABP3: Heart fatty acid-binding protein 3
FDA: Food and Drug Administration
GAP-43: Growth-associated protein 43
LNPs: Lipid-based nanoparticles
LOAD: Late onset AD
LT: Lutein
MNPs: Metal-based nanoparticles
MCI: Mild cognitive impairment
ND: Neurodegenerative diseases
NIR: Phenothiazine-based near-infrared
NfL: Neurofilament light
NFTs: Neurofibrillary tangles
Ng: Neurogranin
NLCs: Nanostructured lipid carriers
NPs: Nanoparticles
PCL: Poly-ε-caprolactone
PEI: Polyethyleneimine
PLA: Polylactide
PLGA: Poly(lactide-co-glycolide)
PNPs: Polymeric nanoparticles
PS1: Presenilin-1
PS2: Presenilin-2
p-tau: Hyperphosphorylated tau
SLNs: Solid lipid nanoparticles
sTREM2: Soluble ectodomain of TREM2
TREM2: Triggering receptor expressed on myeloid cells 2
t-tau: Total tau
USPIONs: Ultrasmall superparamagnetic iron oxide nanoparticles
VILIP-1: Visinin-like protein 1
